# Modulating the electronic structure of a semiconductor to optimize its electrochemiluminescence performance†

**DOI:** 10.1039/c9na00011a

**Published:** 2019-03-27

**Authors:** Xiaohong Wang, Miao Zhang, Xiaolei Huo, Wei Zhao, Bing Kang, Jing-Juan Xu, Hongyuan Chen

**Affiliations:** State Key Laboratory of Analytical Chemistry for Life Science and Collaborative Innovation Center of Chemistry for Life Sciences, School of Chemistry and Chemical Engineering, Nanjing University 210023 China binkang@nju.edu.cn xujj@nju.edu.cn

## Abstract

Electrochemiluminescence (ECL) is a light emission process originating from the energy relaxation of excited chemical states. For semiconducting materials, the ECL performance highly depends on the electronic band structure and the relaxation dynamics of charge carriers in excited states. Even though extensive investigations have been attempted, how the electronic structure relates to and affects the final ECL performance has not been fully understood thus far. Here, using carbon dots (CDs) as a model system, we reported the electrochemiluminescence (ECL) of carbon dots with different nitrogen doping concentrations obtained *via* a hydrothermal method. Nitrogen doping tuned the electronic structure of the carbon dots, resulting in a broadened band gap and slower decay dynamics. These two aspects restrained nonradiative recombination and promoted radiative recombination, which ultimately enhanced the ECL performance.

## Abstract 2

How the band gap and relaxation dynamics of a semiconductor affect its electrochemiluminescence performance was investigated.

## Graphical abstract

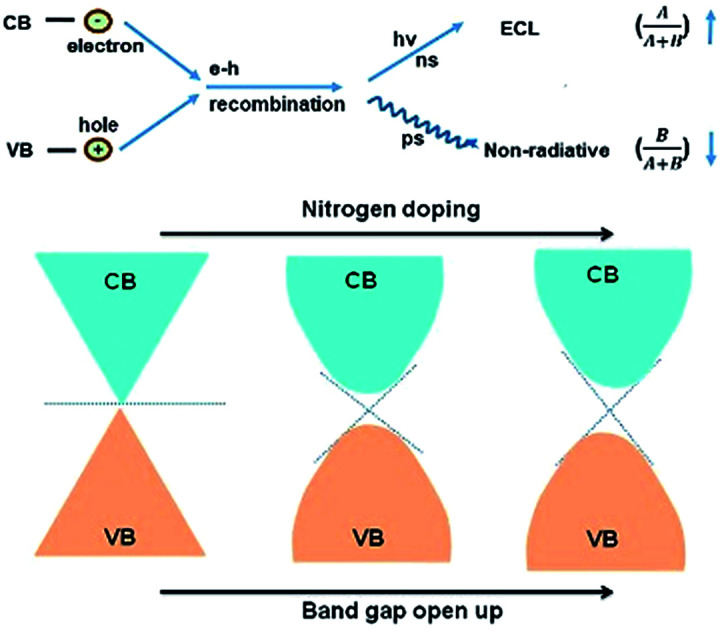

## Introduction

Electrochemiluminescence (ECL) is a light emission process triggered by the energy relaxation of excited chemical states.^1–3^ Its versatility, simplified optical setup, low background signals, high sensitivity and fast sample analysis abilities endow ECL with many advantages from an analytical perspective.^4–6^ For semiconducting materials, ECL emission is caused by electron–hole recombination. Theoretically, tuning the recombination dynamics of electrons and holes can modulate the final ECL performance.^7,8^ In many cases, the ECL emission depends on the surface states of the ECL material, which are closely related to the electronic band structure and the relaxation dynamics of charge carriers in excited states.^9,10^ Revealing the relation between the electronic structure and ECL behavior may provide important insights for a deeper understanding of ECL mechanisms as well as for the design of high performance ECL systems.

Carbon dots (CDs) have been extensively explored as ECL materials, because of the unique properties of their surface states, which are sensitive to doping with other atoms, like nitrogen (N).^8,11^ Heteroatom doping can effectively alter the local electronic and chemical characteristics.^12–15^ For instance, N doping has been proven to improve the ECL emission of CDs through the injection of electrons into the conduction band and the upward shifting of the Fermi energy level, or through the production of surface traps and the further acceleration of electron transfer between the co-reactant and CDs, owing to the electron rich and electron deficient nature of N atoms.^1,8,16,17^ Despite intensive investigations, however, the relation between the electronic structures of materials and their final ECL performance still remain unclear thus far.

In this work, CDs with different N doping concentrations were synthesized *via* a facile hydrothermal method. The nitrogen content in the nitrogen doped carbon dots (NCDs) was changed through the concentration of ammonium hydroxide, and this induced changes in their intrinsic features, especially the luminescence and semiconducting properties. Furthermore, the relationship between the ECL enhancement of CDs and their band gaps and PL lifetimes was studied, and it was found that the ECL performance of CDs could be modulated through the electronic structure. From analysis of the band gaps and lifetimes of excited states, a broader band gap and slower decay dynamics were considered to be two main aspects that enhanced the final ECL performance.

## Results and discussion

**Fig. 1:**
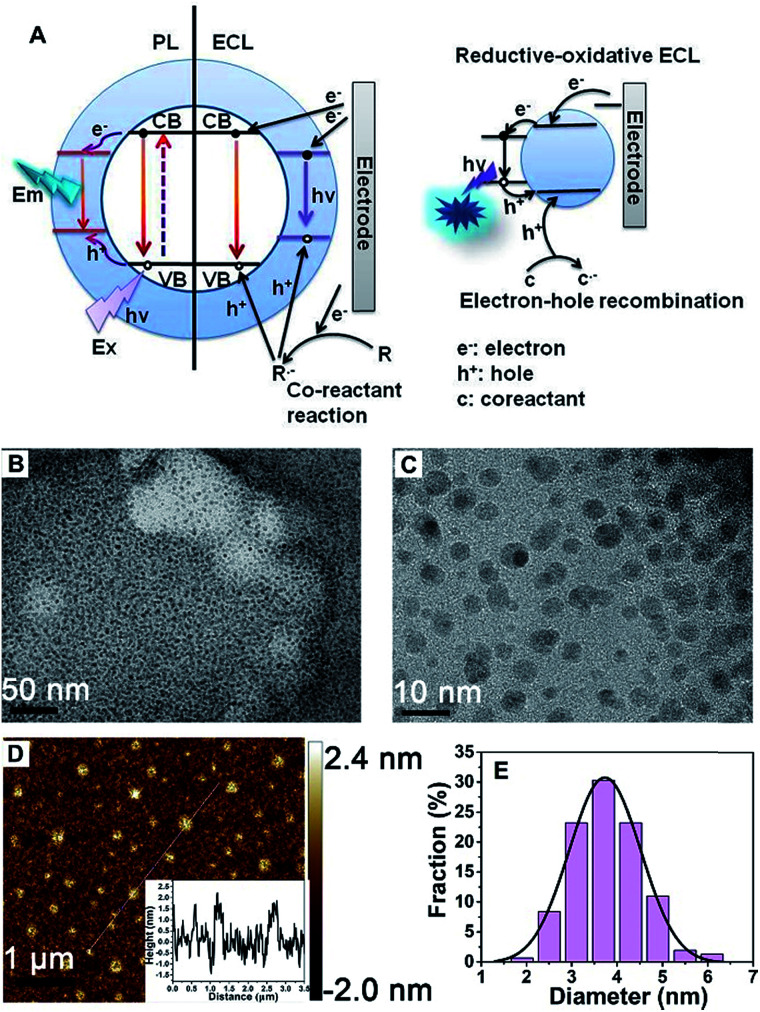
Mechanism details and the morphology of the CDs. (A) A schematic illustration of PL and ECL emission from CDs: a comparison of the PL and ECL mechanisms involving CDs (left) and a schematic mechanism of reductive-oxidative (R–O) ECL from CDs (right). (B, C) Low-magnification and high-magnification TEM images of CDs. (D) An AFM image of CDs deposited on a silicon slice (inset: the height profile along the line shown in the topographic image of the CDs). (E) A particle size distribution histogram from the CDs.

A schematic illustration of the CD-based ECL system is shown in Fig. 1A. Compared to PL, a process that is determined by electron transition between the valence band and conduction band, the ECL process mainly depends on electron–hole recombination at the particle surface. Thus the electronic structure and recombination dynamics, especially with respect to the surface states, are expected to govern the ECL performance.^2^

The morphology of the obtained CDs was observed using transmission electron microscopy (TEM) and atomic force microscopy (AFM). Fig. 1B and C shows typical TEM images of the as-prepared CDs. The as-prepared CDs are nearly spherical, uniform in size and well dispersed, and their thickness is about 2 nm (Fig. 1D). It can be seen from Fig. 1E that the CDs have a mean particle diameter of 3.71 ± 0.2 nm, with a range between 2 nm and 6 nm, as determined from 200 nanoparticle counts. In this system, the sizes of the CDs and NCDs are almost the same (Fig. S1†).

**Fig. 2:**
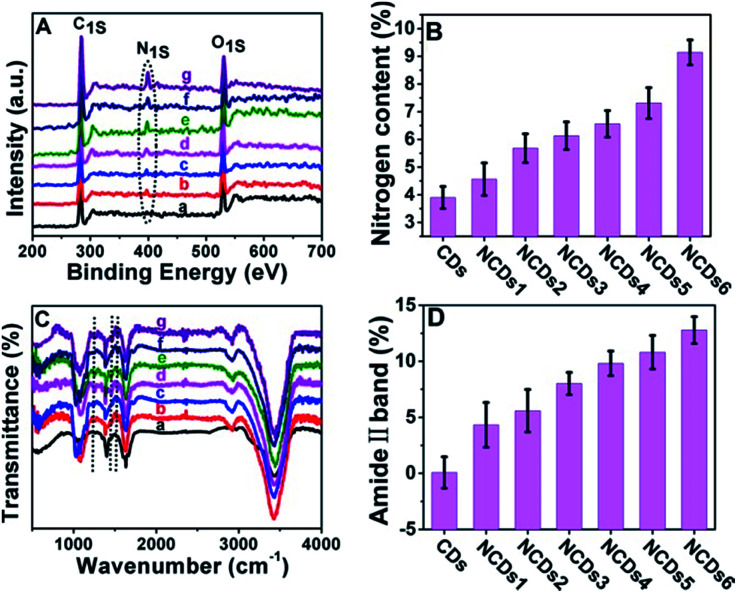
Characterization of the CDs and NCDs1–NCDs6. (A) Full-scan XPS spectra of the CDs and NCDs1–NCDs6 (from (a to g) CDs; NCDs1; NCDs2; NCDs3; NCDs4; NCDs5; NCDs6). The dashed-dotted line shows the N 1s content in the CDs and NCDs1–NCDs6. (B) The nitrogen content of the CDs and NCDs1–NCDs6. (C) FTIR spectra (from (a to g) CDs; NCDs1; NCDs2; NCDs3; NCDs4; NCDs5; NCDs6). The three dashed-dotted lines correspond, from left to right, to the C–N amine stretching vibrations, the amine III band from C–N bonds and the amide II band from N–H bonds, respectively. (D) The relative intensity of the amide II bands from N–H bonds (the internal standard is C=O) of the CDs and NCDs1–NCDs6.

In our current system, the electronic structure of the CDs was tuned by N doping into the CDs. The doping content was characterized *via* X-ray photoelectron spectroscopy (XPS). The N signal can barely be seen in the initial CDs (Fig. S2†); the N peak intensity became stronger and stronger as the ammonia concentration increased (Fig. 2A and B). In full scan XPS spectra of NCDs1–NCDs6, the N peak intensity got stronger and stronger with an increase in the N content in the NCDs (Fig. S3†). The doping of N into the CDs was also confirmed *via* Fourier transform infrared spectroscopy (FTIR; Fig. 2C); with an increase in the N content, the intensities of the amide II band from N–H bonds, the C–N amine stretching vibration, and the amine III band from C–N bonds, corresponding to 1532 cm^−1^, 1250 cm^−1^ and 1445 cm^−1^, respectively, obviously strengthen.^18^ Taking the C=O peak at 1636 cm^−1^ as an internal reference, the amide II band at 1532 cm^−1^ representing the nitrogen content increased (Fig. 2D). From the XPS and FTIR results, the obtained NCDs have multiple C- and N-containing groups, which manifests that nitrogen not only functionalized on the surface of the NCDs, but it was also doped into the lattices of the carbon skeleton.

**Fig. 3:**
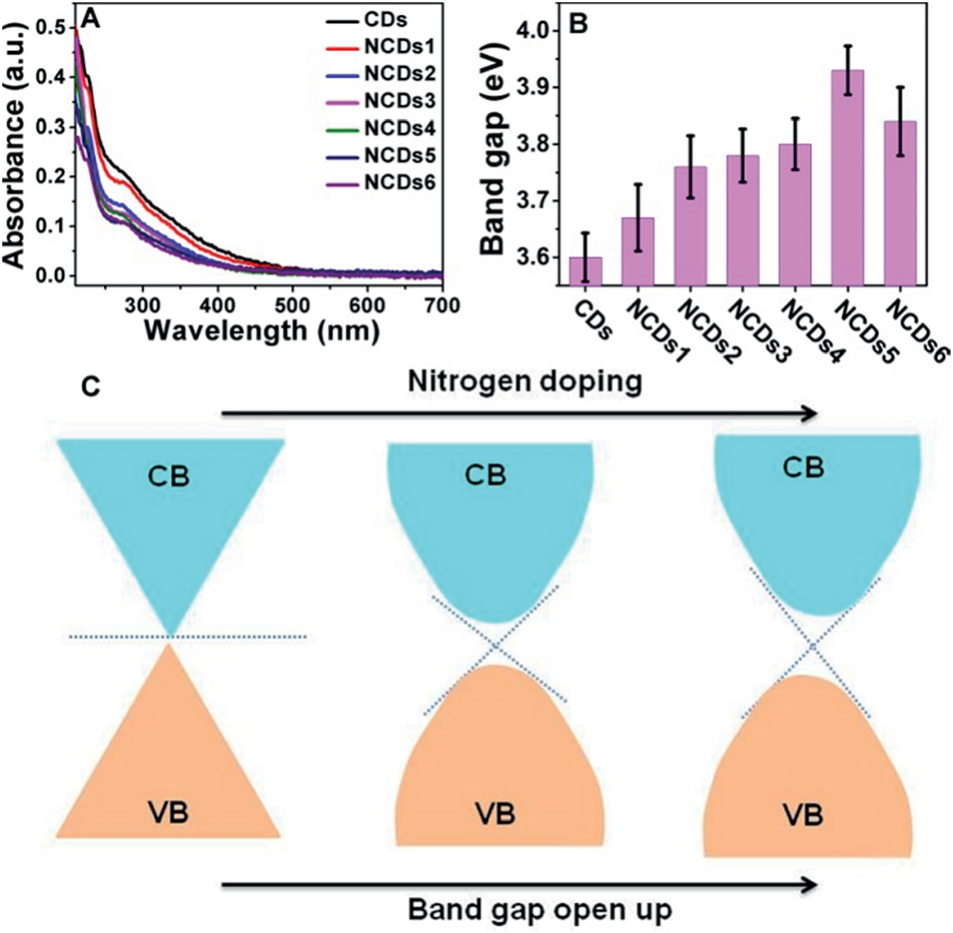
The energy structures of the CDs and NCDs1–NCDs6. (A) UV-vis absorbance spectra of the CDs and NCDs1–NCDs6. (B) The band gaps of the CDs and NCDs1–NCDs6. (C) A simulated band gap map, where CB stands for conduction band and VB stands for valence band.

As shown in Fig. 3A, with an increase of N doping, the UV-vis peaks remained relatively close, and the position of the peaks is about 280 nm. As shown in Fig. S4,† a correlation curve relating to the band gap can be made from ultraviolet absorption spectra; the relationship between the optical absorption coefficient of a semiconductor and the band gap is:(*αhυ*)^1/*n*^ = *C*(*hυ* − *E*_g_)where *α* is the sample absorbance, *C* is a constant, *hυ* is the photon energy, and *E*_g_ is the band gap of the material. In this equation, the value of *n* depends on the nature of the electronic transition. *n* = 1/2 and *n* = 2 signify direct transition and indirect transition, respectively.^19^ In this method, the optical band gap value, *E*_g_, of the semiconductor material was calculated through the extrapolation of the linear part of the curve from a plot of (*αhυ*)^1/2^*vs. hυ* to where it intersected with the *x*-axis.^20^

As shown in Fig. 3C, the pure CDs with a graphene-like structure can be considered as a semiconductor with zero band gap. In practical samples, the surface layers of CDs are usually oxidized, forming a small band gap.^21,22^ N doping into the CDs resulted in electron groups like amino groups forming on the surface, further opening up the band gap.^23,24^ However, when the doping amount is too large, too many intermediate bands may be formed between the VB and CB, which can weaken the semiconducting characteristics. The doped N atoms modified the electronic structure and induced changes in the Fermi levels, creating a dopant effect and opening the band gap of the NCDs.^21,24^ A high nitrogen content endows the NCDs with a strong electronic donating nature and leads to a larger band gap, directly causing a decrease in non-radiative recombination in the CDs.^25^

**Fig. 4:**
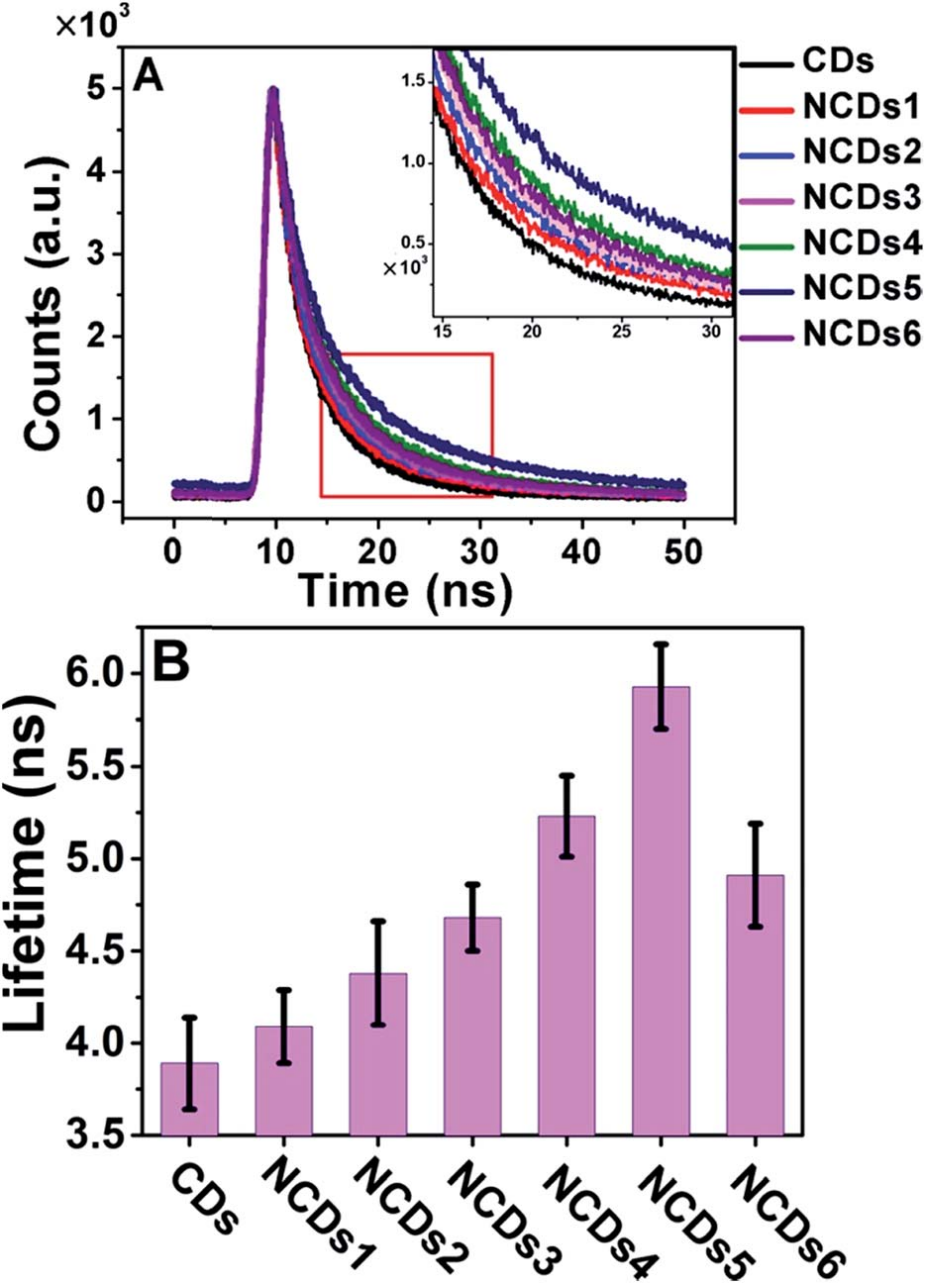
PL properties of the CDs and NCDs1–NCDs6. (A) Time-resolved fluorescence decay (*λ*_ex_ = 365 nm) measured at 440 nm. (B) The PL lifetimes of the CDs and NCDs1–NCDs6.

A change in the band structure usually affects the transition dynamics, *i.e.* the lifetimes of excited state electrons. We therefore measured the fluorescence lifetimes of the CDs and NCDs1–NCDs6 with excitation and emission wavelengths of 365 and 440 nm, respectively (Fig. 4A).

As the nitrogen content increased from NCDs1 to NCDs6, the fluorescence lifetime increased from 3.81 ns to a maximum of 5.93 ns for NCDs5, before decreasing to 4.91 ns for NCDs6 (Fig. 4B). Excessive N doping in NCDs6 led to a decrease in the lifetime from 5.93 ns to 4.91 ns. This tendency is consistent with the decrease in the band gap shown Fig. 3B, and is possibly due to the inactivation of zigzag-edge sites and effective energy transfer between N atoms and the NCDs.^16,24^ For semiconducting materials, the lifetime of excited state electrons largely influences their luminescence properties, including PL and ECL.

**Fig. 5:**
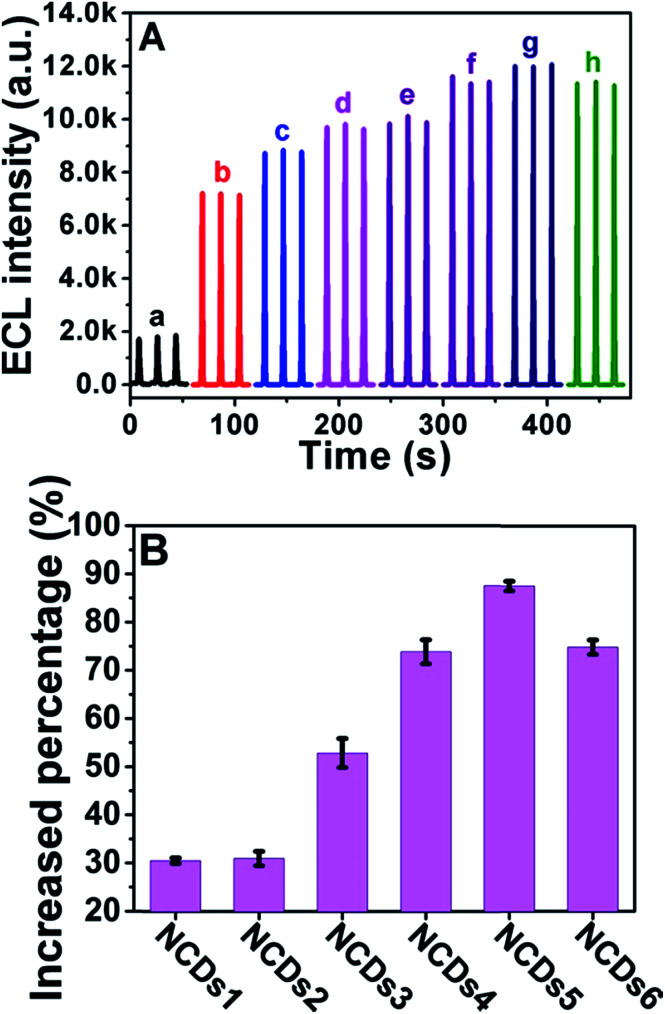
The ECL response of the CDs and NCDs1–NCDs6. (A) ECL emission with 100 mM S_2_O_8_^2−^ under continuous cyclic scanning from −1.8 V to 0 V in 100 mM PBS (pH 7.4, containing 100 mM KCl) over 20 cycles. The voltage of the photomultiplier tube (PMT) was set to 700 V (from (a to h) GCE; CDs; NCDs1; NCDs2; NCDs3; NCDs4; NCDs5; NCDs6). (B) The percentage increase in ECL intensity compared to the CDs.

We then investigated the ECL performance of the initial CDs and N doped NCDs under identical experimental conditions. Fig. 5 exhibits the ECL spectra of NCD-modified GCEs in the presence of 100 mM K_2_S_2_O_8_ in 100 mM PBS (pH = 7.4) containing 100 mM KCl as the supporting electrolyte at a scan rate of 200 mV s^−1^ between −1.8 V and 0 V (*vs.* Ag/AgCl). As shown in Fig. 5A, the ECL intensity kept increasing with an increase in the N doping concentration from NCDs1 to NCDs5, with a maximum intensity from NCDs5, and then it decreased slightly for NCDs6. When the N doping concentration becomes too high, the band structure of the NCDs may be distorted and the intensity of the ECL may also decrease.

**Fig. 6:**
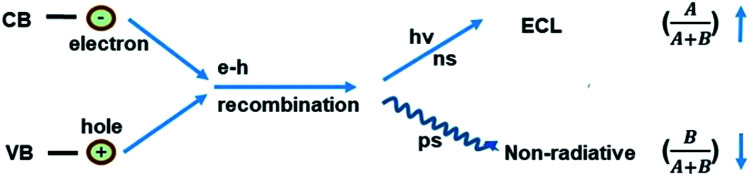
A schematic illustration of electron–hole recombination.

Doping N atoms has been proposed as a method to improve electrochemical performance *via* engineering the band gap.^7,25^ Our above results have demonstrated that N doping into CDs broadened the band gap, prolonged the lifetime of excited states and finally promoting the ECL performance. To profoundly understand how the band gap and excited state lifetimes related to the ECL behavior, we considered these processes from the viewpoint of fundamental transition dynamics. For a simplified two energy level system, like the VB and CB of a semiconductor, the excited electrons could undergo relaxation through two typical paths, *i.e.* radiative and non-radiative. For PL lifetime measurements, the decay dynamics of the PL intensity depend on both of the above-mentioned processes. Therefore, the time-dependent PL intensity *I*(*t*) can be written as a double exponential function:^26,27^
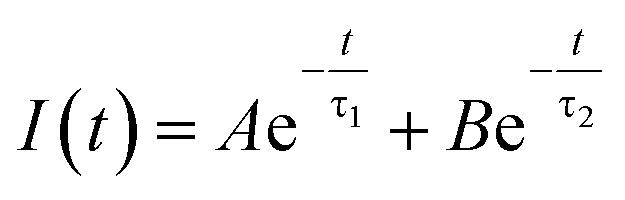
where *τ*_1_ and *τ*_2_ are the time constants of the radiative and non-radiative relaxation processes, respectively, and *A* and *B* are constants.^28,29^ Considering the relaxation rate of both radiative and non-radiative processes, the total weighted mean PL lifetime (*τ*_total_) could be expressed as:
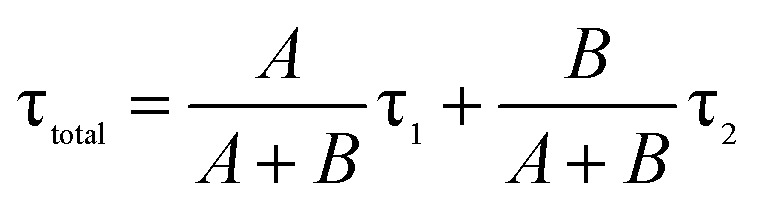
where *A*/(*A* + *B*) and *B*/(*A* + *B*) represent the weights of *τ*_1_ and *τ*_2_, respectively. Since the non-radiative process (∼ps) is much faster than the radiative process (∼ns), thus for a mixed process, a more radiative process usually leads to a longer lifetime (*τ*_total_) and a more non-radiative process leads to a shorter lifetime (*τ*_total_). For an ECL process, even with a different excitation mechanism (Fig. 6), its properties are still governed by the above-mentioned two fundamental processes, since the final luminescence also originates from excited state electron relaxation (also called electron–hole recombination). According to the energy gap law, the rate of non-radiative relaxation decreases exponentially along with an increase in the band gap.^17,24^ Thus, a broadened band gap would restrain non-radiative relaxation and promote the radiative relaxation process, meanwhile prolonging the lifetime. For an ECL process, these aspects would ultimately enhance the ECL performance.

From the perspective of band gaps and lifetimes, as nitrogen doping into CDs increases, reduced non-radiative recombination and enhanced radiative recombination lead to the ECL intensity increasing.^7^ Excessive N doping in NCDs causes a slight decrease in ECL, possibly because excessive N doping results in self-quenching functionality or because the structure of the NCDs becomes destroyed.^16^

## Conclusions

In conclusion, using carbon dots (CDs) as a model system, we demonstrated the modulation of ECL behavior through tuning the electronic structure of an electrode material. A series of CDs with different nitrogen doping concentrations were prepared *via* a hydrothermal method. The nitrogen doping into the CDs changed their electronic structure, leading to a broadened band gap and slower decay dynamics. These two aspects resulted in a decrease in nonradiative recombination and an increase in radiative recombination, which remarkably promoted the final ECL performance. A theoretical model based on the energy gap law and relaxation dynamics was proposed to explain the above-mentioned process. For semiconducting materials, the ECL performance highly depends on the electronic band structure and the relaxation dynamics of charge carriers in excited states. The concept of modulating ECL behavior *via* the electronic structure of a material proposed here may provide profitable insights into understanding the mechanism of the ECL process, as well as into the design of high performance ECL systems.

## Conflicts of interest

There are no conflicts to declare.

## Supplementary Material

NA-001-C9NA00011A-s001
