# Supplementary material for: Modulating the electronic structure of a semiconductor to optimize its electrochemiluminescence performance
Source: Nanoscale Adv. 2019 Mar 27;1(5):1965–9. doi: 10.1039/c9na00011a (PMC9419581; doi:10.1039/c9na00011a)
Supplement: NA-001-C9NA00011A-s001 [file NA-001-C9NA00011A-s001.pdf]

Electronic Supplementary Material (ESI) for Nanoscale Advances.
This journal is © The Royal Society of Chemistry 2019

Enhanced electrochemiluminescence of carbon dots by tuning its electronic structure

Xiao-Hong Wang, Miao Zhang, Xiao-Lei Huo, Wei Zhao, Bing Kang, * Jing-Juan Xu, * Hong-Yuan Chen

State Key Laboratory of Analytical Chemistry for Life Science and Collaborative Innovation Center of Chemistry for Life Sciences, School of Chemistry and Chemical Engineering, Nanjing University, 210023, China. E-mail: binkang@nju.edu.cn; xujj@nju.edu.cn

Table of Contents:

1. Experimental Section

1.1. Experimental Section 2

1.2. Preparation of N-Doped carbon dots 2

1.3. Characterizations 2

1.4. Electrochemical Measurements 2

2. Supporting Figures 3

Figure S1. Morphology of NCDs1-NCDs6 4

Figure S2. Characterizations of CDs 5

Figure S3. XPS survey spectra of CDs and NCDs1- NCDs6 6

Figure S4. Optical properties of CDs 7

1. Experimental Section

1.1. Reagents and Materials. $NH_3 \cdot H_2O$ (25-28 wt %) were purchased from Tianjin Yongda Chemical Reagent Factory (Tianjin, China). Other reagents were purchased from Beijing Chemical Reagent Factory (Beijing, China). Other chemicals used in this study were analytical grade. Green soy bean was obtained from local marketplace (Nanjing, China). All solutions were prepared with ultra-pure water, purified by a Millipore-Q System (18.2 M cm).

1.2. Preparation of N-Doped carbon dots.

1 g green soy bean was placed in 18 mL ultrapure water. Next, the mixture was transferred into an 25 mL Teflon-lined stainless-steel autoclave and heated at 200 ºC for 12 h in a hot air oven, then cooled down to room temperature. CDs were prepared by 18 mL water and 1 g green soy bean under 200 ºC for 12 h. The different nitrogen content of CDs were prepared by mix 1 mL ammonium hydroxide with 17 mL ultrapure water (the all solution 18 mL) named as NCDs1, and so on 3 mL ammonium hydroxide named as NCDs2, 5 mL ammonium hydroxide named as NCDs3, 7 mL ammonium hydroxide named as NCDs4, 9 mL ammonium hydroxide named as NCDs5, 11 mL ammonium hydroxide named as NCDs6. The solution was filtered with 220 nm membrane, and the larger carbon particles were filtered out several times. The CDs was further purified by a dialysis membrane (1000 da) to remove the residual ammonium hydroxide for 2 days. The CDs were concentrated and dried in a vacuum (60 ºC).

1.3. Characterizations. The ECL emission measurements were carried out on a Home-made ECL analyzer (Remax Electronic Instrument Limited Co., Xi'an, China, 350 nm~650 nm). The UV-vis absorption spectra were performed on a Shimadzu UV-3600 UV-vis-NIR spectrophotometer (Shimadzu Co.). Transmission electron microscopy (TEM) image was obtained through a JEOL model 2000 instrument operating at 200 kV accelerating voltage.

1.4. Electrochemical Measurements. ECL measurement was performed on MPI-E model ECL analyzer using the as prepared CDs/nafion modified on a glassy carbon electrode (GCE) as the working electrode. Briefly, GCE were polished in sequential order with 1.0- and 0.05-μm alumina slurry and then thoroughly washed with ultrapure water and ethanol, in turn. After the electrodes were polished and dried with nitrogen gas, 8 μL of the as-prepared CDs solution was deposited on the polished GCE and naturally dried, and then the electrode was dropped with 0.05 wt % nafion soulution. In the ECL test, platinum wire and Ag/AgCl electrode were used as counter electrode and the reference electrode respectively, and the GCE modified with materials ware used as working electrode. The GCE electrode was conducted in 100 mM PBS (pH 7.4) with $K_2S_2O_8$ (100 mM) and KCl (100 mM) and scanned from 0 to −1.8 V for 200 mV/s, and the photomultiplier tube (PMT) was set at 700 V.

2. Supporting Figures

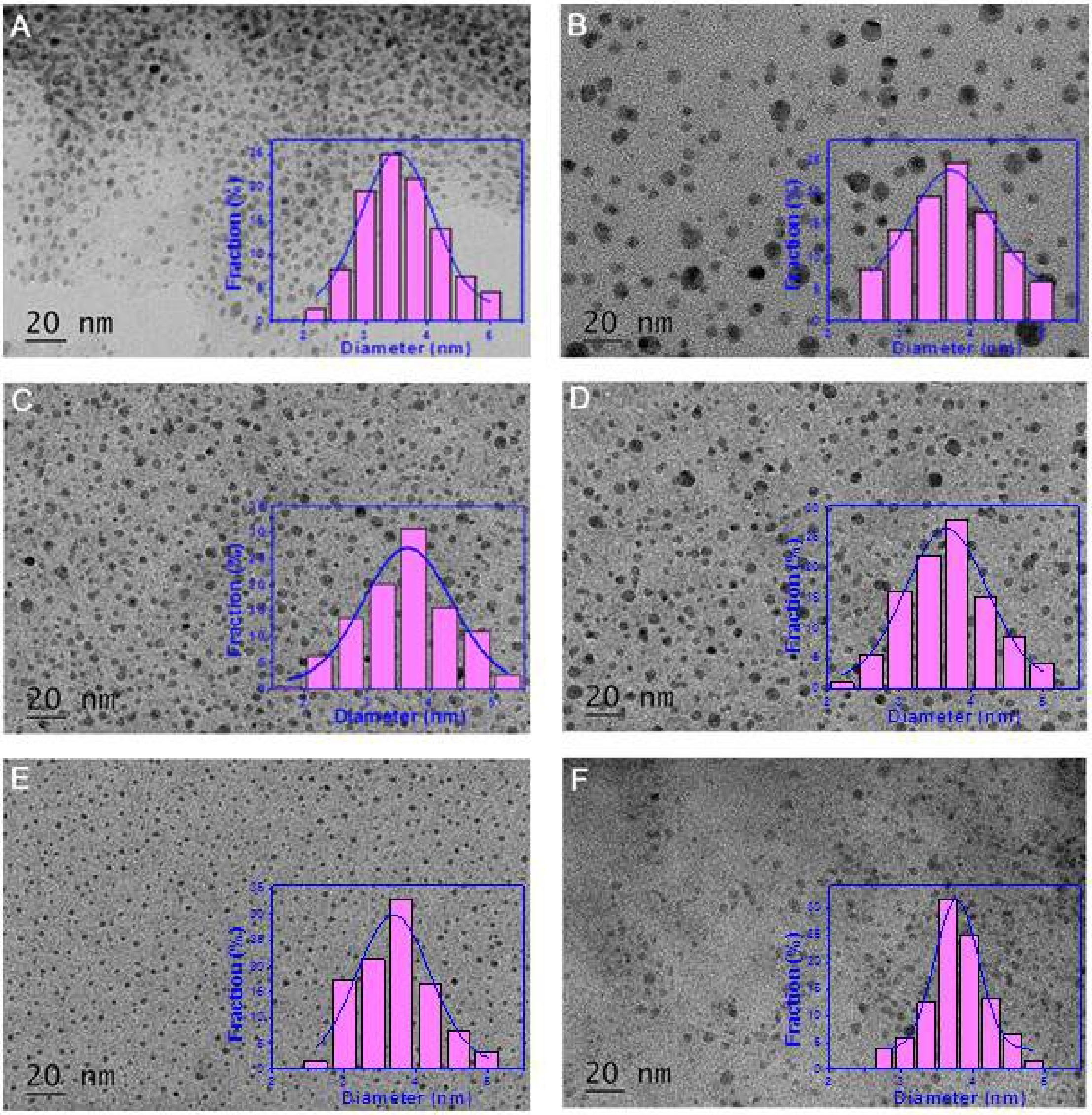

Figure S1. Morphology of NCDs1-NCDs6. (A) TEM images of NCDs1 (a mean particle diameter of 3.58±0.2 nm), (B) TEM images of NCDs2 (a mean particle diameter of 3.73±0.2 nm), (C) TEM images of NCDs3 (a mean particle diameter of 3.68±0.2 nm), (D) TEM images of NCDs4 (a mean particle diameter of 3.67±0.2 nm), (E) TEM images of NCDs5 (a mean particle diameter of 3.71±0.2 nm), (F) TEM images of NCDs6 (a mean particle diameter of 3.77±0.2 nm). (inset: The particle size distribution histogram of CDs).

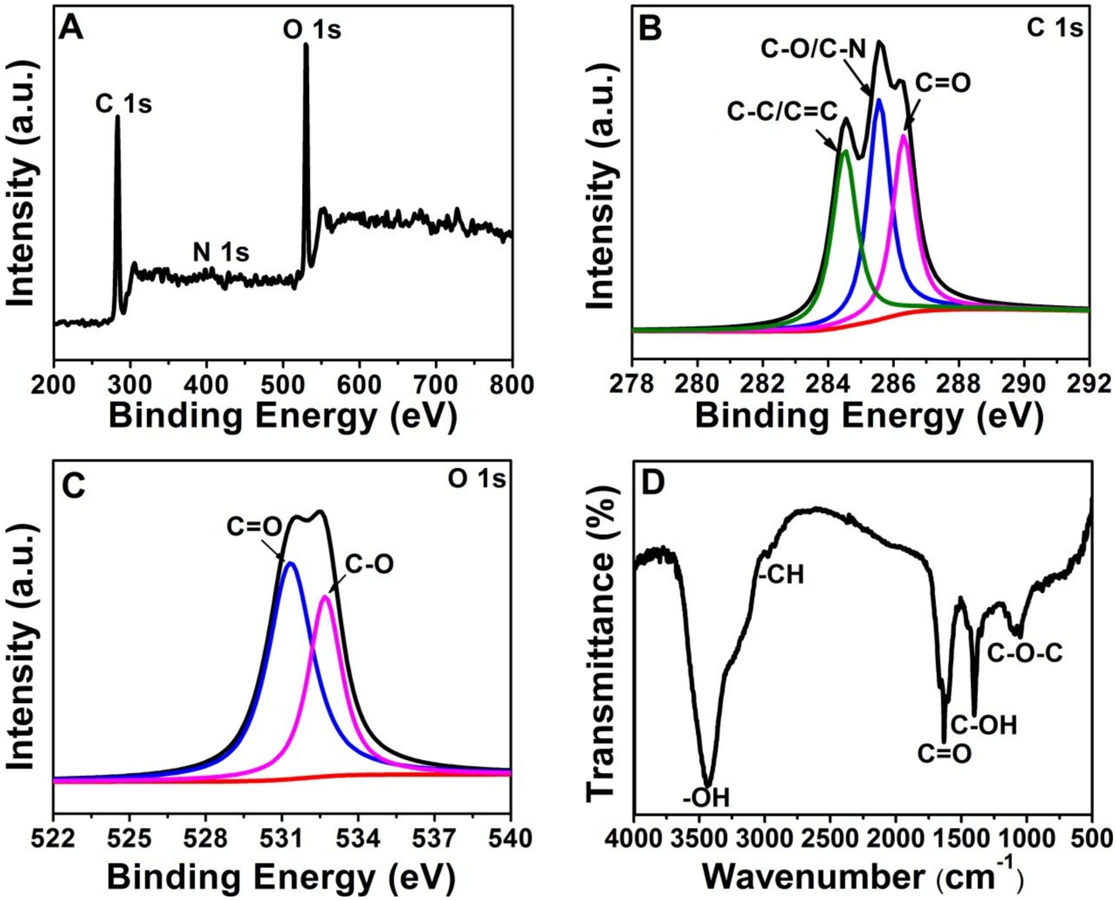

Figure S2. Characterizations of CDs. (A) XPS spectra and high-resolution XPS spectra of (B) C 1s, (C) O 1s and (D) FT-IR spectra of CDs.

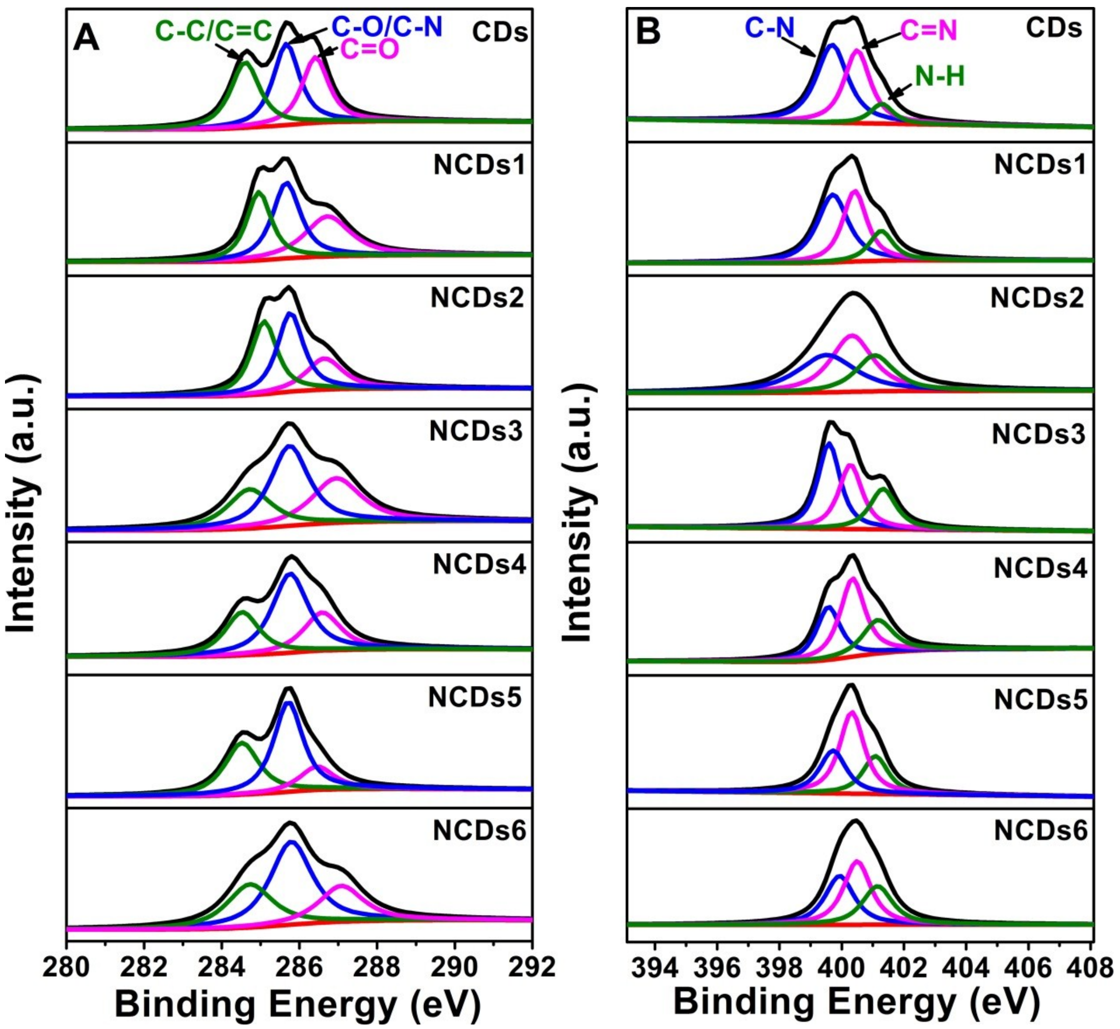

Figure S3. XPS survey spectra of CDs and NCDs1- NCDs6. (A) XPS C 1s spectra, and (B) XPS N 1s spectra.

The electron transfer between CDs and the electrode surface was facilitated on the GC electrode. The possible ECL process of cathode ECL of CDs is elucidated as follows[1]:

$$CDs + e \longrightarrow CDs^{\bullet-} \quad (1)$$

$$S_2O_8^{2-} + e \longrightarrow SO_4^{2-} + SO_4^{\bullet-} \quad (2)$$

$$CDs^{\bullet-} + SO_4^{\bullet-} \longrightarrow CDs^{*} + SO_4^{2-} \quad (3)$$

$$CDs^{*} \longrightarrow CDs + h\nu \quad (4)$$

As the electrode potential is scanned to more negative, CDs gets electrons to form $CDs^{\bullet-}$, and $S_2O_8^{2-}$ get the strong oxidant sulfuric acid free radical ions released by electrons. $SO_4^{\bullet-}$ get electrons from CDs free radicals, which become the excited state CDs^{*}, which release energy due to excited state CDs* fall to ground state CDs. The results show that CDs can be used as an efficient light luminophore to produce ECL.

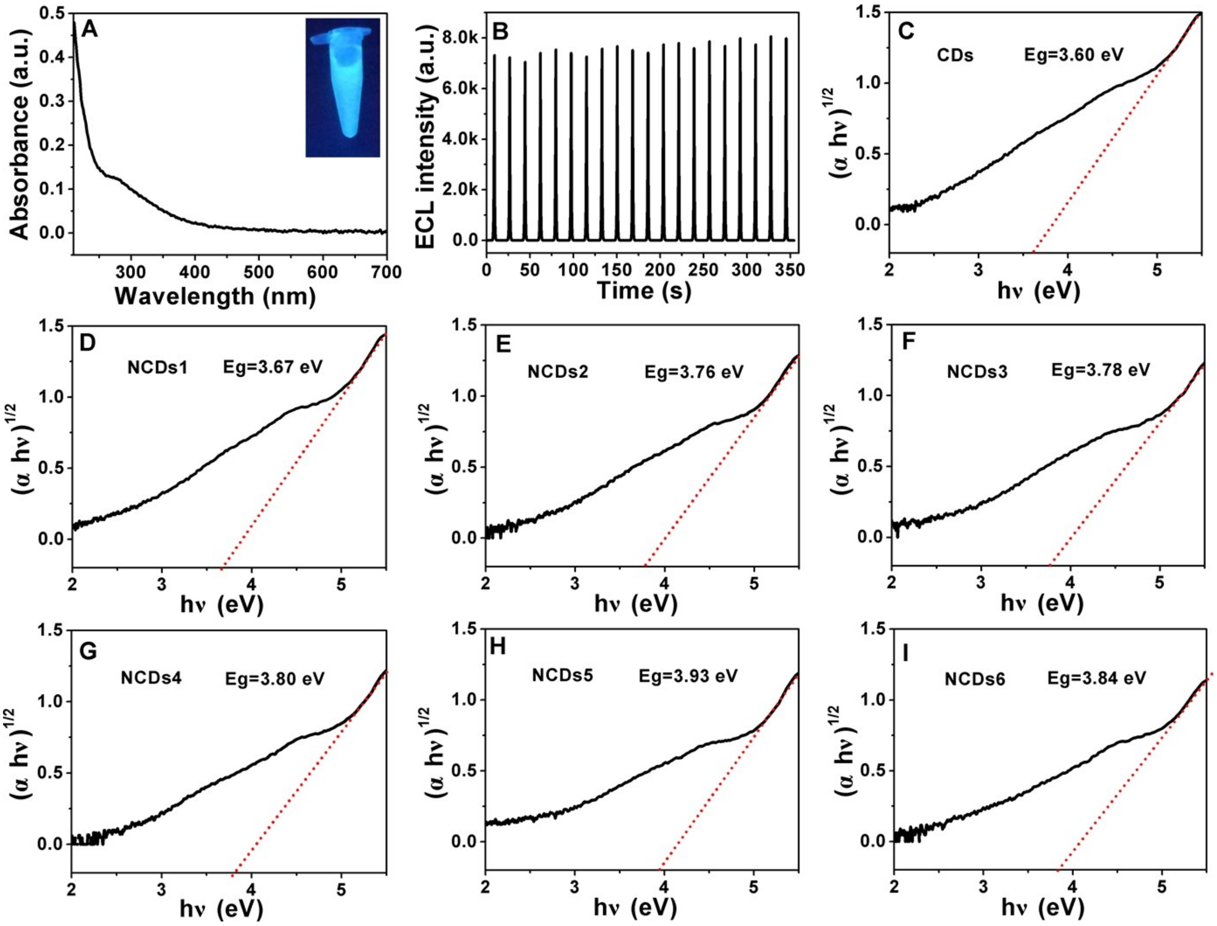

Figure S4. Optical properties of CDs. (A) UV-visible absorbance spectra of CDs in aqueous solution. Inset: digital photograph of CDs solution under UV light. (B) ECL emission from CDs/nafion with 100 mM $S_2O_8^{2-}$ under continuous cyclic scans from -1.8 V to 0 V in 100 mM PBS (containing 100 mM KCl) for 20 cycles. The voltage of the photomultiplier tube (PMT) was set as 700 V. (C-I) The corresponding Tauc plots of CDs and NCDs1-NCDs6.

1. T. Han, T. Yan, Y. Li, W. Cao, X. Pang, Q. Huang and Q. Wei, *Carbon*, 2015, **91**, 144-152.
